# Regulation of Plant Growth, Photosynthesis, Antioxidation and Osmosis by an Arbuscular Mycorrhizal Fungus in Watermelon Seedlings under Well-Watered and Drought Conditions

**DOI:** 10.3389/fpls.2016.00644

**Published:** 2016-05-11

**Authors:** Yanling Mo, Yongqi Wang, Ruiping Yang, Junxian Zheng, Changming Liu, Hao Li, Jianxiang Ma, Yong Zhang, Chunhua Wei, Xian Zhang

**Affiliations:** ^1^College of Horticulture, Northwest A&F UniversityYangling, China; ^2^Hanzhong City Agro-technology Extension CenterHanzhong, China; ^3^Shangluo UniversityShangluo, China

**Keywords:** arbuscular mycorrhizal fungus, drought stress, plant growth, photosynthesis, antioxidant system, osmotic adjustment, watermelon

## Abstract

Drought stress has become an increasingly serious environmental issue that influences the growth and production of watermelon. Previous studies found that arbuscular mycorrhizal (AM) colonization improved the fruit yield and water use efficiency (WUE) of watermelon grown under water stress; however, the exact mechanisms remain unknown. In this study, the effects of *Glomus versiforme* symbiosis on the growth, physio-biochemical attributes, and stress-responsive gene expressions of watermelon seedlings grown under well-watered and drought conditions were investigated. The results showed that AM colonization did not significantly influence the shoot growth of watermelon seedlings under well-watered conditions but did promote root development irrespective of water treatment. Drought stress decreased the leaf relative water content and chlorophyll concentration, but to a lesser extent in the AM plants. Compared with the non-mycorrhizal seedlings, mycorrhizal plants had higher non-photochemical quenching values, which reduced the chloroplast ultrastructural damage in the mesophyll cells and thus maintained higher photosynthetic efficiency. Moreover, AM inoculation led to significant enhancements in the enzyme activities and gene expressions of superoxide dismutase, catalase, ascorbate peroxidase, glutathione reductase, and monodehydroascorbate reductase in watermelon leaves upon drought imposition. Consequently, AM plants exhibited lower accumulation of MDA, H_2_O_2_ and $O_{2}^{-}$ compared with non-mycorrhizal plants. Under drought stress, the soluble sugar and proline contents were significantly increased, and further enhancements were observed by pre-treating the drought-stressed plants with AM. Taken together, our findings indicate that mycorrhizal colonization enhances watermelon drought tolerance through a stronger root system, greater protection of photosynthetic apparatus, a more efficient antioxidant system and improved osmoregulation. This study contributes to advances in the knowledge of AM-induced drought tolerance.

## Introduction

Drought is well known as a significant environmental problem that restricts plant growth and crop yield worldwide. Due to climate change, drought is expected to worsen in the near future, particularly in arid and semiarid regions (Gong et al., 2013). Drought affects many aspects of plants, inhibiting photosynthesis, limiting water uptake, damaging plasma membranes, and ultimately resulting in decreased growth (Talbi et al., 2015). Although plants can avoid the damage caused by drought stress through a variety of self-response mechanisms including increased root growth, enhanced antioxidant enzyme activities and the accumulation of compatible solutes, etc., their ability to do so is limited. Therefore, considerable efforts in crop management practices are being encouraged to overcome water deficit stress and enhance drought tolerance, such as the application of various types of exogenous substances and beneficial microorganisms such as arbuscular mycorrhizal (AM) fungi (Huang et al., 2011).

Arbuscular mycorrhizal fungi are important soil microorganisms that are widely distributed in almost all terrestrial ecosystems and form symbiotic relationships with the roots of nearly 80% of all plant species (Yang Y. et al., 2014). Symbiosis with AM fungi can positively enhance plant nutrient acquisition (Cartmill et al., 2012), promoting plant growth and favoring survival under both biotic and abiotic stress conditions without harming the environment (Tian et al., 2013; Song et al., 2015). Such an eco-friendly and effective biological technique to enhance plant resistance to adverse environmental conditions, particularly drought stress, has received increasing attention from crop scientists in recent years (Maya and Matsubara, 2013). Many studies have investigated and proved the possibility of improving plant drought tolerance by AM inoculation (Huang et al., 2011). The improved adaptation of plants inoculated with AM to drought stress conditions was thought to be linked to a combination of physical, physiological, and cellular effects (Ruiz-Lozano, 2003), which commonly include the following: enriched soil moisture and soil properties (Yooyongwech et al., 2013), enhanced water and nutrient uptake and transpiration (Smith et al., 2009; Yang Y. et al., 2014), induction of plant growth promoting hormonal signals (Yang Y. et al., 2014), and increased antioxidase activity and photosynthetic rate (Huang et al., 2011). However, the precise mechanisms involved are still under debate and depend on the plant species involved (Zhang et al., 2015).

Watermelon [*Citrullus lanatus* (Thunb.) Matsum. & Nakai] is an important summer crop of high economic and nutritious value worldwide (Guo et al., 2011), but a high water-consuming plant due to its large leaf area and fruit that contains a high water content. Thus, its cultivation is heavily dependent on irrigation, especially during the fruit development stage (Kaya et al., 2003). Limited water availability results in the delay of vine elongation and leaf expansion, and long-term drought stress causes flower and fruit dropping, thereby leading to low fruit yield. According to the FAO, China, with approximately 1.8 million ha under watermelon cultivation in 2013, is currently the top global producer of watermelon (FAO of the United Nations, 2015). However, most Chinese watermelon varieties are intolerant of drought stress as a consequence of long-term selection and breeding pressure for good performance under irrigated field conditions (Zhang et al., 2011). Moreover, the major production area of watermelon is distributed in Northern China, which is a typical arid and semi-arid region that is usually subjected to drought stress and poor water management. Therefore, agricultural practices are necessary in these areas to enable watermelon plants to withstand drought (Omirou et al., 2013). It has been reported that the use of AM fungi is able to improve fruit yield and WUE of watermelon plants under insufficient irrigation conditions (Kaya et al., 2003; Omirou et al., 2013). This suggests an acceptable and promising method to mitigate the adverse effects of water stress on watermelon production in these areas. However, little is known about the mechanisms responsible for improving drought tolerance by inoculating this cucurbit plant species with AM fungi. Therefore, the AM fungal effects on the drought tolerance of watermelon plants were investigated here and the corresponding mechanisms were explored from both physiological and molecular aspects, focusing particularly on photosynthesis, the antioxidant systems, osmotic adjustment and stress-responsive gene expressions.

## Materials and Methods

### Plant and Fungal Material

A drought-sensitive watermelon variety (Y34) was provided by the Watermelon and Melon Research Group at Northwest A&F University, Yangling, Shaanxi, China. AM inocula of *Glomus versiforme* were obtained from the Beijing Academy of Agriculture and Forestry Sciences, Beijing, China. The mycorrhizal inoculum was a stock culture of sand, spores (with approximately 730 spores per 10 g of air-dried soil), hyphae and colonized clover (*Trifolium repens* L.) root fragments (with an average colonization rate of 90%).

### Experimental Design and Water Treatments

Experiments were conducted in a greenhouse at Northwest A&F University, Yangling (34°283′N, 108°067′E), China. The treatments consisted of two levels of irrigation and two AMF conditions in a randomized block design, namely: (I) well-watered plants without AM inoculation (WW-NM); (II) well-watered plants with AM inoculation (WW+M); (III) drought-stressed plants without AM inoculation (DS-NM); and (IV) drought-stressed plants with AM inoculation (DS+M). Each treatment included three replications.

Seeds were sterilized with 2% sodium hypochlorite for 10 min, pre-soaked at 25°C for 4–6 h and maintained in the dark at 30°C until germination. Germinated seeds with similar germination lengths were sown in plastic pots [10 cm (H) × 7 cm (W) × 8.5 cm (D)] with one seed per pot. The growth medium used in the experiments was a 1:1 (v/v) autoclaved mixture of sand and commercial peat-based compost (Shaanxi Yufeng Seed Industry Co., LTD). The properties of the soil mix were as follows: pH, 7.57; water field capacity, 52.21%; organic matter content, 3.42 g kg^-1^; available N, P, and K, 73.72, 12.23, and 118.74 mg kg^-1^, respectively. Each plastic pot was filled with 355 g of air-dried soil mix and 10 g AM inoculum (for mycorrhizal plants) or the same weight of autoclaved inoculum (for non-mycorrhizal plants). Mycorrhizal inocula were placed directly below the watermelon seeds at the time of sowing. The seedlings were pre-cultured under natural light in a greenhouse where the relative humidity was 65–95%, and the temperature was 28–35°C in the daytime and 16–20°C at night. Before the treatment initiation, all seedlings were well watered each day and were fertilized weekly with 1/2 strength Hoagland’s solution (pH 6.5). When the plants had 4–5 true leaves, the seedlings were randomly selected and subjected to the water treatments. The WW treatments were controlled at 75 ± 5% field capacity (FC), while the DS treatments were watered to 30 ± 5% of field capacity (FC). The soil water status was measured daily in the evening, and the amount of water lost was supplied to each pot to maintain the designated soil moisture content. Water loss was evaluated gravimetrically by weighing the pots, and the amount of water loss in each pot was the difference between a re-watered pot and the actual weight of the pot 24 h later. Aluminum foil was used to cover the pots to minimize water loss (Gong et al., 2013). After 12 days of treatment, samples were harvested with at least three biological replicates. Leaf sampling was randomly conducted on the second uppermost fully expanded leaves from the plants in each treatment group.

### Observation of AM Development and Plant Growth Measurements

A segment collected from the middle part of nine fine roots from each treatment was carefully washed, cut into 1-cm long fragments and cleared with 10% (w/v) KOH, then stained with 0.05% (w/v) trypan blue to investigate AM colonization. AM colonization (%) was calculated as 100 × root length infected/root length observed (Wu and Xia, 2006). Examination of AM development was performed using a microscope (Olympus BH2, Japan) equipped with a Nikon DXM1200 digital camera.

On the last day of the experiment, 12 seedlings were harvested per treatment, carefully washed and divided into shoots and roots. After recording the plant height, leaf number, and root length, the tissues were oven-dried for 72 h at 80°C to obtain the dry mass. The root/shoot ratio was calculated as follows: Root/shoot ratio = Root dry mass/Shoot dry mass.

### Leaf Relative Water Content (RWC) and Chlorophyll Concentration

Six pots were randomly selected in each treatment to determine the leaf RWC and chlorophyll concentration. Leaf RWC was determined as described by Barrs and Weatherley (1962). The chlorophyll concentration (Chlorophyll a+b) was quantified according to Lichtenthaler and Wellburn (1982).

### Chloroplast Ultrastructure

Three leaves per treatment were collected to examine the chloroplast ultrastructure of the mesophyll cells. Tissues were sliced into 2 mm × 4 mm pieces and the specimens were prepared following the description of Wang et al. (2012). The chloroplast ultrastructure was observed and photographed under a JEM-1230 transmission electron microscope (JEOL Ltd., Tokyo, Japan) at 80 kV.

### Initial Rubisco Activity, and Photosynthetic and Chlorophyll Fluorescence Parameters

Initial Rubisco activity was assayed following the method of Zhang L. et al. (2013). Photosynthetic parameters were determined using a portable LI-6400 photosynthesis system (Li-6400; Li-Cor, Lincoln, NE, USA) equipped with an LED red/blue light source (6400-02B). The CO_2_ concentration in the chamber was 400 ± 10 μmol mol^-1^, the photosynthetic photon flux density was 500 μmol m^-2^ s^-1^, and an air flow rate of 500 μmol s^-1^ and a temperature of 28 ± 2°C were used. The photosynthesis rate (*P*_n_) and transpiration rate (*T*_r_) were recorded automatically. Instantaneous water use efficiency (iWUE) was calculated as *P*_n_/*T*_r_ (Mielke et al., 2005). Chlorophyll fluorescence measurements were carried out with a portable PAM-2500 fluorometer (Walz, Germany) coupled to computer-operated PAM-control software (PAMWin 3.0). The maximum photochemical efficiency of PSII (*F*v/*F*m, *F*v = *F*_m_–*F*_0_), actual photochemical efficiency of PSII [ΦPSII = (F_m_′- F_s_)/F_m_′], electron transport rate (ETR, ETR = ΦPSII × 0.5 × PPFD × 0.84), photochemical quenching [qP = (F_m_′- F_s_)/(F_m_′- F_0_′)], and non-photochemical quenching [NPQ = (F_m_ -F_m_′)/F_m_′] were calculated according to Maxwell and Johnson (2000).

### Lipid Peroxidation, Fydrogen Peroxide (H_2_O_2_) and Superoxide Anion Radical ($O_{2}^{-}$) Determination

The level of lipid peroxidation was estimated as equivalents of malondialdehyde (MDA) using the thiobarbituric acid (TBA) method according to Guo et al. (2012). H_2_O_2_ and $O_{2}^{-}$ were extracted and measured according to the method of Bai et al. (2010). The generation of H_2_O_2_ and $O_{2}^{-}$ was also visually detected in the sampled leaves using 3, 3-diaminobenzidine (DAB) and nitroblue tetrazolium (NBT), respectively, as the substrate. For the histochemical staining of H_2_O_2_, leaf disks (1.5 cm in diameter) were placed in a solution consisting of 1 mg mL^-1^ 3, 3-diaminobenzidine (DAB, pH 5.5) for 6 h after a slight vacuum infiltration, rinsed in a large quantity of a 95% (v/v) ethanol solution for 10 min, and then photographed with a digital camera (Canon EOS 5D; Canon Inc., Tokyo, Japan; Chen et al., 2013). For the histochemical staining of $O_{2}^{-}$, leaf disks were incubated in a 25 mM K-HEPES buffer (pH 7.8) containing 0.1 mg mL^-1^ NBT at 25°C for 4 h under dark conditions. Afterward, the foliar disks were boiled in a 95% (v/v) ethanol solution and photographed as described above (Kadkhodaie et al., 2014).

### Antioxidant Enzyme Extraction and Activity Assays

Frozen leaf samples (0.5 g) were homogenized in 8 ml of cold 50 mM PBS (pH 7.8) containing 0.1 mM EDTA and 1% (m/v) polyvinylpolypyrrolidone in a chilled mortar. The homogenate was centrifuged at 12,000 *g* for 20 min at 4°C, and the resulting supernatant was used to assay the following enzyme activities.

Superoxide dismutase (SOD) activity was measured by monitoring the inhibition of the photochemical reduction of NBT as described by Bai et al. (2010). Catalase (CAT), ascorbate peroxidase (APX), glutathione reductase (GR), monodehydroascorbate reductase (MDHAR), and dehydroascorbate reductase (DHAR) activities were determined following the method of Guo et al. (2012). CAT activity was assayed by monitoring the decrease in absorbance at 240 nm because of H_2_O_2_ decomposition (extinction coefficient of 39.4 mM^-1^ cm^-1^). The 1-ml reaction mixture contained 50 mM PBS (pH 7.0), 10 mM H_2_O_2_ and 20 μl enzyme extract. The reaction was initiated by adding H_2_O_2_. For APX activity, we determined the decrease in absorbance at 290 nm by tracking the oxidation of reduced ascorbate (ASA; extinction coefficient of 2.8 mM^-1^ cm^-1^) in a 1-ml reaction mixture containing 50 mM Hepes-KOH (pH 7.6), 0.1 mM EDTA-Na_2_, 0.5 mM ASA, 1 mM H_2_O_2_, and 20 μl supernatant. The reaction was initiated by adding H_2_O_2_. GR activity was measured from the absorbance change at 340 nm because of NADPH oxidation (extinction coefficient of 6.22 mM^-1^ cm^-1^). The 1-ml reaction mixture contained 100 mM Tris-HCl buffer (pH 7.5), 0.1 mM NADPH, 1 mM EDTA-Na_2_, 0.25 mM oxidized glutathione (GSSG) and 20 μl enzyme extract. The reaction was initiated by adding NADPH. MDHAR activity was assayed by monitoring the decrease in absorbance at 340 nm due to NADH oxidation (extinction coefficient of 6.2 mM^-1^ cm^-1^). The 1-ml reaction mixture contained 50 mM Hepes-KOH (pH 7.6), 2.5 mM ASA, 0.1 mM NADH, 0.5 U ASA oxidase, and 20 μL enzyme extract. The reaction was initiated by adding ASA oxidase. DHAR activity was determined by monitoring the increase in absorbance at 265 nm because of ASA formation (extinction coefficient of 14 mM^-1^ cm^-1^). The 1-ml reaction mixture contained 50 mM Hepes-KOH (pH 7.6), 2.5 mM reduced glutathione (GSH), 0.1 mM EDTA-Na2, 0.2 mM dehydroascorbate (DHA), and 20 μl enzyme extract. The reaction was initiated by adding DHA.

### ASA and GSH Determination

The reduced ASA and DHA contents were determined following the method of Logan et al. (1998). The reduced GSH and GSSG contents were measured according to Guo et al. (2012). The DHA content was estimated by subtracting the value for ASA from the total ASA, and the GSH content was the difference between total GSH and GSSG.

### Soluble Sugar Content and Proline Determination

Total soluble sugar and free proline contents were determined using the anthrone sulphuric acid method and the ninhydrin method, respectively, according to Gao (2000).

### RNA Extraction and mRNA Expression Analysis

Ten watermelon genes of interest, i.e., Rubisco small subunit (*RBCS*), Rubisco large subunit (*RBCL*), pheide a oxygenase (*PAO*), pheophytin pheophorbide hydrolase (*PPH*), *Cu-Zn SOD*, *CAT*, c*APX*, c*GR*, *MDHAR*, and *DHAR* were previously searched from the Cucurbit Genomics Database^1^ to perform qRT-PCR. The corresponding specific primers are detailed in Supplementary Table S1. Total RNA was extracted using TRIzol Reagent (Invitrogen, USA) and treated with RNase-free DNase I (Invitrogen, Los Angeles, CA, USA) to remove genomic DNA. Reverse transcription was conducted using 1 μg Dnase-treated RNA. The qRT-PCR was carried out using an iCycler iQ TM Multicolor PCR Detection System (Bio-Rad, Hercules, CA, USA) and an SYBR Premix ExTaq II (2x) Kit (Takara) under cycling conditions of 95°C for 3 min; 95°C for 30 s; 58°C for 30 s (40 cycles); and 72°C for 30 s. These qRT-PCR experiments were performed in three replicates, on the basis of three separate RNA extracts from three leaf samples. The relative expressions of the mRNAs were calculated using the 2^-ΔΔCT^ method (Livak and Schmittgen, 2001), and the watermelon β-actin gene was used as the reference (Kong et al., 2014).

### Statistical Analysis

Data were analyzed using two-way analysis of variance (ANOVA) with “inoculation” and “watering” as main fixed factors. *T*-test (*P* < 0.05) was performed to compare differences between inoculation treatments under the same water treatment. Percentage values were arcsin transformed before statistical analysis. Statistical analysis was performed using the PASW Statistics 18.0 program. Values are presented as the means ± standard deviation (SD) of 3–12 replicate samples.

## Results

### AM Colonization and Plant Growth

**FIGURE 1:**
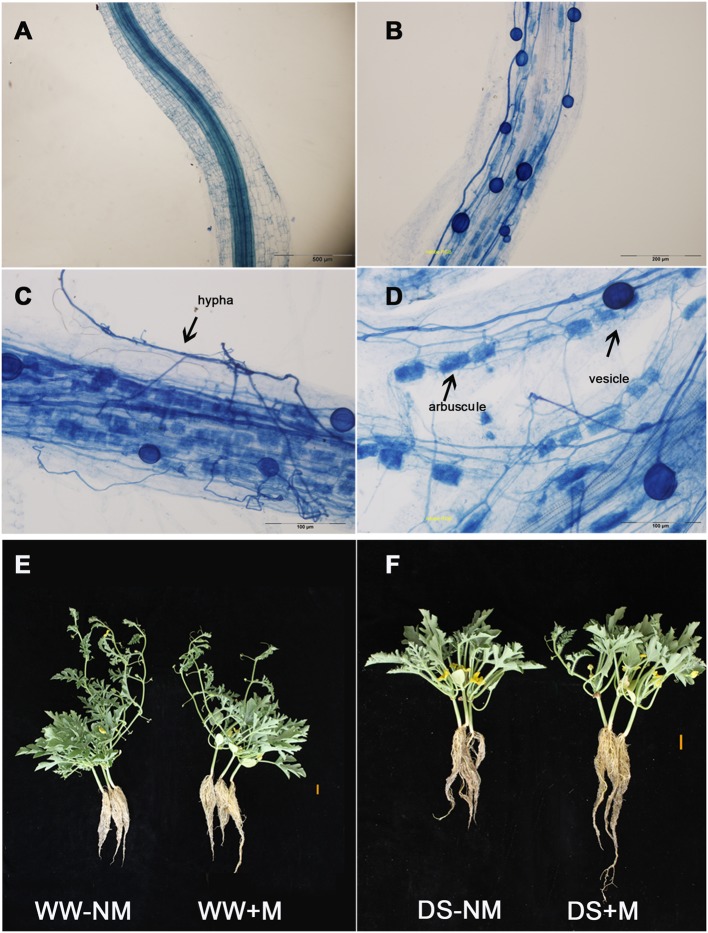
**The development of the arbuscular mycorrhizal (AM) fungus in watermelon roots (as revealed by trypan blue staining) and the morphological characteristics of the mycorrhizal (+M) and non-mycorrhizal (-NM) watermelon seedlings grown under well-watered (WW) and drought-stressed (DS) conditions. (A)** Root cells without AM inoculation (bars 500 μm). **(B)** Root cells with AM inoculation (bars 200 μm). **(C)** Photographs showing the hypha (bars 100 μm). **(D)** Photographs showing the vesicle and arbuscules (bars 100 μm). **(E)** Well-watered plants with and without AM inoculation (bars 2 cm). **(F)** Drought-stressed plants with and without AM inoculation (bars 2 cm).

No AM root colonization was detected in the non-inoculated watermelon plants, while good symbiosis was established in the inoculated plants, with a colonization rate above 75% under both WW and DS conditions (**Figures 1A,B**; **Table 1**). After the AM fungal inoculation, a large number of hyphae were observed in the epidermic and cortical cells of the AM plant roots, and typical vesicles as well as arbuscules were detected in most of the cortex cells (**Figures 1C,D**). Twelve days of drought stress significantly decreased the plant height, leaf number, and biomass production of the watermelon seedlings (**Figures 1E,F**; **Table 1**). Mycorrhizal colonization improved plant growth, especially the root growth under the water deficit condition, as indicated by higher values of almost all parameters in inoculated plants than in non-inoculated plants. Under WW conditions, mycorrhizal colonization did not positively influence any shoot growth index but did stimulate both the root dry mass accumulation and the root/shoot ratio. Interactions between watering and AM inoculation were significant for plant height (*P* < 0.05), root length and leaf number (*P* < 0.01).

**Table 1:** Mycorrhizal root colonization rate and growth of mycorrhizal (+M) and non-mycorrhizal (-NM) watermelon seedlings grown under well-watered (WW) and drought-stressed (DS) conditions.

Water condition	Inoculation	AM colonization (%)	Plant height (cm)	Root length (cm)	Leaf number (No./plant)	Dry weight (g)		Root/shoot ratio
						Shoot	Root	
WW	-NM	0 ± 0.00b	32.44 ± 3.23a	17.14 ± 1.11a	12.13 ± 0.74a	2.78 ± 0.12a	0.28 ± 0.02b	0.10 ± 0.01b
	+M	79.07 ± 7.33a	30.77 ± 2.91a	17.76 ± 1.25a	11.73 ± 0.70b	2.81 ± 0.11a	0.37 ± 0.03a	0.13 ± 0.01a
DS	-NM	0 ± 0.00b	8.09 ± 1.73b	14.50 ± 0.83b	6.60 ± 0.63b	1.29 ± 0.08b	0.21 ± 0.02b	0.16 ± 0.01b
	+M	76.63 ± 7.84a	9.73 ± 1.00a	19.25 ± 1.48a	7.07 ± 0.26a	1.42 ± 0.06a	0.27 ± 0.02a	0.19 ± 0.02a
Significance								
Watering (W)		ns	^∗∗^	ns	^∗∗^	^∗∗^	^∗∗^	^∗∗^
Inoculation (I)		^∗∗^	ns	^∗∗^	ns	^∗^	^∗∗^	^∗∗^
W × I		ns	^∗^	^∗∗^	^∗∗^	ns	ns	ns

*T-test was performed to compare differences between inoculation treatments under the same water treatment. Different letters following the values indicate significant differences between treatments at *P* < 0.05. Two-way ANOVA output: ns, not significant; ^∗^*P* < 0.05, ^∗∗^*P* < 0.01. Percentage values were arcsin transformed before statistical analysis. Values represent the means ± SD (*n* = 9 for AMF colonization, 12 for growth parameters)*.

### Leaf Water Status, Chlorophyll Concentration, and Chloroplast Ultrastructure

**FIGURE 2:**
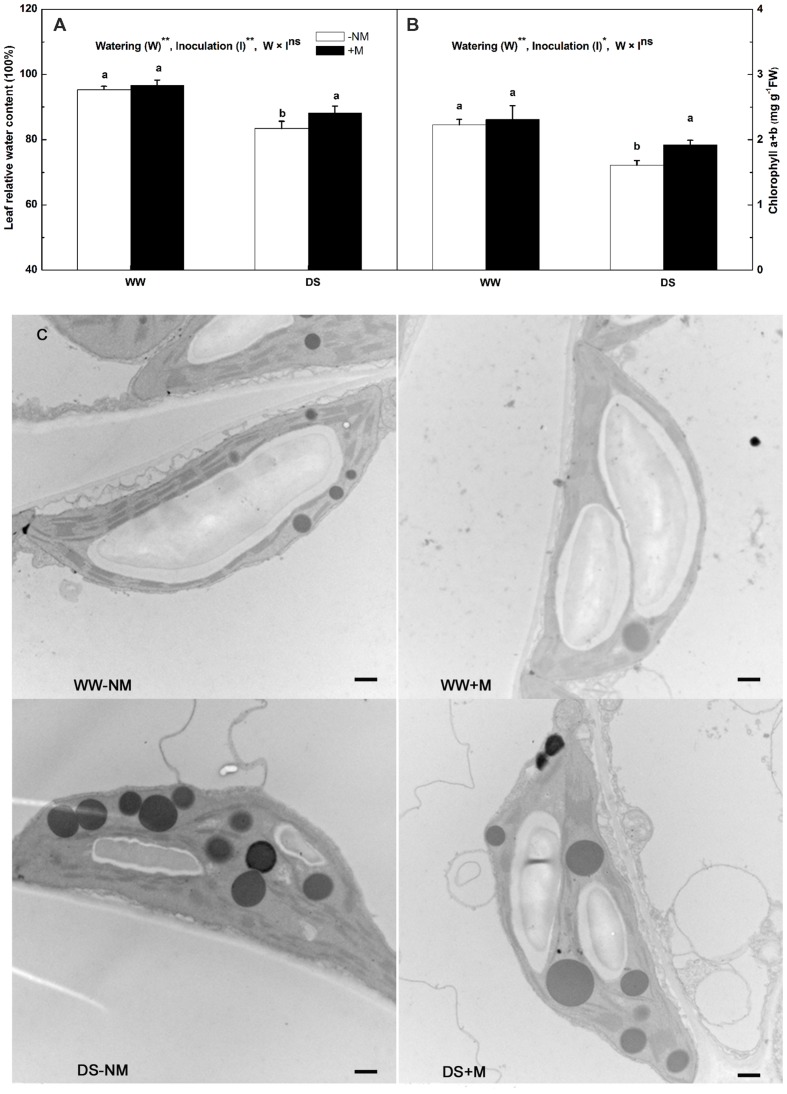
**Leaf relative water content (RWC; **A)**, chlorophyll concentration **(B)** and chloroplast ultrastructure **(C)** of mesophyll cells in mycorrhizal (+M) and non-mycorrhizal (-NM) watermelon seedlings grown under WW and DS conditions**. Scale bars: **(C)** 500 nm. *T*-test was performed to compare differences between inoculation treatments under the same water treatment. Different letters following the values indicate significant differences between treatments at *P* < 0.05. Two-way ANOVA output: ns, not significant; ^∗^*P* < 0.05, ^∗∗^*P* < 0.01. Data represent the means ± SD of six replicates.

The leaf RWC in the watermelon plants, which was approximately 95% under WW conditions, was not affected by *G. versiforme* colonization (**Figure 2A**). Drought stress markedly reduced the leaf RWC; this was observed to a greater extent in the non-inoculated than in the inoculated plants. Under WW conditions, the chlorophyll concentration was maintained at high levels irrespective of the AM pretreatment, but was significantly reduced by drought stress, i.e., to 83.17 and 72.30% of their respective controls in the stressed mycorrhizal and non-mycorrhizal plants, respectively (**Figure 2B**). A decrease in the chlorophyll content may reflect pigment destruction in the chloroplast; thus, we examined the chloroplast ultrastructure of the watermelon leaves to determine the impairment of the photosynthetic system. The chloroplasts of plants that received a normal water supply exhibited good grana and stroma thylakoid arrangement, with large starch grains but a few number of small osmiophilic globules. By contrast, the stressed plants showed obvious ultrastructural changes and damage, including a disaggregated thylakoid system, reduced levels of grana stacking, fewer starch grains and an accumulation of large osmiophilic globules. Further, the alterations were more drastic in the stressed non-mycorrhizal plants than in the stressed mycorrhizal plants (**Figure 2C**).

### Initial Rubisco Activity, and Photosynthetic and Chlorophyll Fuorescence Parameters

The leaf transpiration rate, instantaneous WUE, initial Rubisco activity and NPQ values were similar between the mycorrhizal and non-mycorrhizal seedlings under WW conditions, but higher levels of *P*_n_, *F*v/*F*m, ΦPSII, ETR, and qP were observed in the former compared with the latter seedlings (**Table 2**). Drought stress inhibited the *P*_n_, *T*_r_, initial Rubisco activity, *F*v/*F*m, ΦPSII, ETR, and qP, but increased the iWUE and NPQ in the watermelon seedlings. Mycorrhizal inoculation alleviated the negative effects of water stress on *P*_n_, initial Rubisco activity, *F*v/*F*m, ΦPSII, ETR, and qP, and enhanced iWUE and NPQ, but had no influence on the *T*_r_ compared with the non-inoculated treatment. Interactions between watering and AM inoculation were significant for *P*_n_, iWUE and NPQ (*P* < 0.05).

**Table 2:** Leaf photosynthetic parameters, initial Rubisco activity and leaf chlorophyll fluorescence parameters of mycorrhizal (+M) and non-mycorrhizal (+NM) watermelon seedlings grown under WW and DS conditions.

Water condition	Inoculation	*P*_n_ (μmol CO_2_ m^-2^ s^-1^)	*T*_r_ (mmol H_2_O m^-2^ s^-1^)	iWUE (μmol CO_2_ mmol H_2_O)	Initial Rubisco activity (μmol min^-1^ g^-1^FW)	*F*v/*F*m	ΦPSII	ETR	qP	NPQ
WW	NM	16.82 ± 0.36b	3.95 ± 0.09a	4.26 ± 0.08a	178.25 ± 11.78a	0.77 ± 0.01b	0.50 ± 0.00b	63.78 ± 0.37b	0.76 ± 0.01b	0.40 ± 0.02a
	M	17.50 ± 0.65a	4.04 ± 0.15a	4.33 ± 0.03ba	200.10 ± 18.46a	0.79 ± 0.01a	0.54 ± 0.01a	68.78 ± 0.67a	0.79 ± 0.02a	0.41 ± 0.02a
DS	NM	8.40 ± 0.76b	1.34 ± 0.09a	6.29 ± 0.52b	75.60 ± 9.80b	0.69 ± 0.02b	0.41 ± 0.02b	52.26 ± 2.13b	0.70 ± 0.01b	0.50 ± 0.03b
	M	11.22 ± 0.75a	1.51 ± 0.17a	7.44 ± 0.45a	106.76 ± 13.44a	0.72 ± 0.00a	0.46 ± 0.01a	58.02 ± 0.90a	0.75 ± 0.01a	0.55 ± 0.02a
Significance										
Watering (W)		^∗∗^	^∗∗^	^∗∗^	^∗∗^	^∗∗^	^∗∗^	^∗∗^	^∗∗^	^∗∗^
Inoculation (I)		^∗∗^	ns	^∗^	^∗^	^∗∗^	^∗∗^	^∗∗^	^∗∗^	^∗∗^
W × I		^∗^	ns	^∗^	ns	ns	ns	ns	ns	^∗^

*T-test was performed to compare differences between inoculation treatments under the same water treatment. Different letters following the values indicate significant differences between treatments at *P* < 0.05. Two-way ANOVA output: ns, not significant; ^∗^*P* < 0.05, ^∗∗^*P* < 0.01. Values represent the means ± SD (*n* = 5)*.

### Lipid Peroxidation and $O_{2}^{-}$ and H_2_O_2_ Generation

**FIGURE 3:**
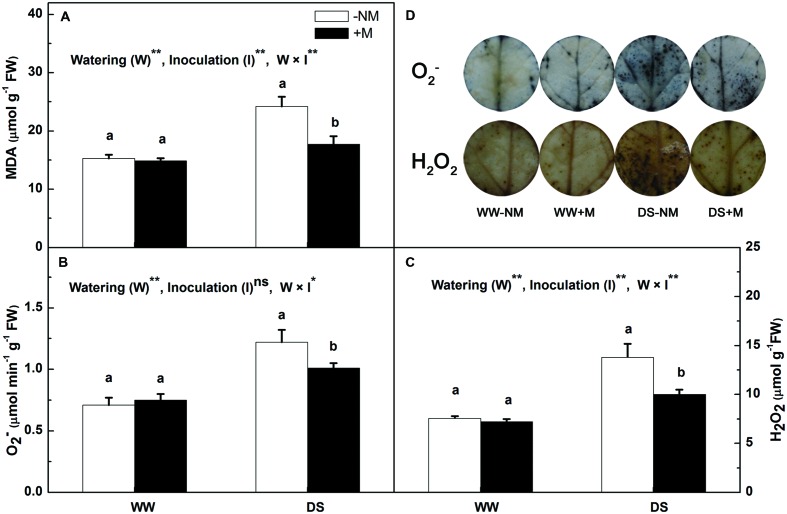
**Malondialdehyde (MDA) content **(A)**, superoxide anion radical ($O_{2}^{-}$) content **(B)**, hydrogen peroxide (H_2_O_2_) content **(C)**, and histochemical staining **(D)** of $O_{2}^{-}$ and H_2_O_2_ in the leaves of mycorrhizal (+M) and non-mycorrhizal (-NM) watermelon seedlings grown under WW and DS conditions**. *T*-test was performed to compare differences between inoculation treatments under the same water treatment. Different letters following the values indicate significant differences between treatments at *P* < 0.05. Two-way ANOVA output: ns, not significant; ^∗^*P* < 0.05, ^∗∗^*P* < 0.01. Data represent the means ± SD of three replicates.

In the well-watered control plants, the leaf MDA, $O_{2}^{-}$, and H_2_O_2_ concentrations remained at low levels regardless of AM inoculation (**Figure 3**). After 12 days of drought stress, the MDA content in the leaf increased considerably, i.e., 1.58-fold higher than the control value (**Figure 3A**). Mycorrhizal colonization significantly reduced the MDA content, which was 26.89% lower than in the drought stress treatment without AM inoculation. The drought treatment also promoted the generation of both $O_{2}^{-}$ and H_2_O_2_, but this effect was more notable in the non-inoculated seedlings relative to the inoculated seedlings (**Figures 3B,C**). This was further confirmed by the *in situ* NBT and DAB staining, where the extent of intensive accumulation of $O_{2}^{-}$ as blue spots and the accumulation of H_2_O_2_ as dark brown spots was considerably less in the DS+M treatment compared with the DS-NM treatment (**Figure 3D**).

### Antioxidant Enzyme Activities and Non-enzymatic Antioxidant Contents

**FIGURE 4:**
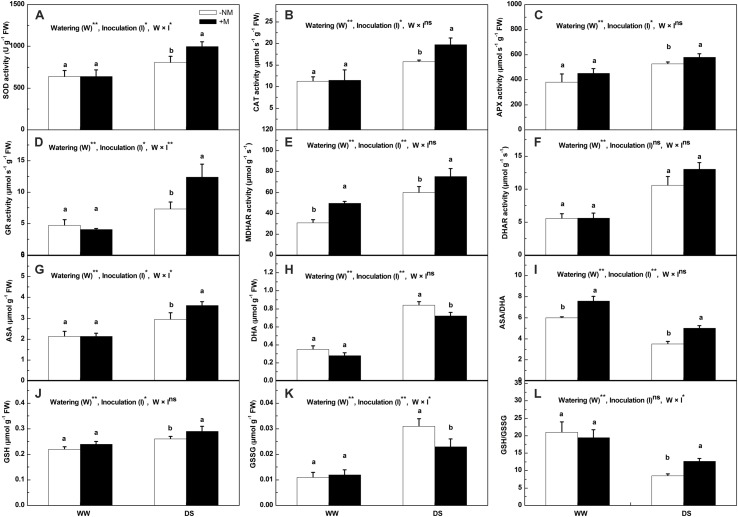
**Antioxidant enzymes activities and antioxidant contents in mycorrhizal (+M) and non-mycorrhizal (-NM) watermelon seedlings grown under WW and DS conditions. (A)** superoxide dismutase (SOD), **(B)** catalase (CAT), **(C)** ascorbate peroxidase (APX), **(D)** glutathione reductase (GR), **(E)** monodehydroascorbate reductase (MDHAR), **(F)** dehydroascorbate reductase (DHAR), **(G)** reduced ascorbate (ASA), **(H)** dehydroascorbate (DHA), **(I)** the ratio of reduced and oxidized ascorbate (ASA/DHA), **(J)** reduced glutathione (GSH), **(K)** oxidized glutathione (GSSG), **(L)** the ratio of reduced and oxidized glutathione (GSH/GSSG). *T*-test was performed to compare differences between inoculation treatments under the same water treatment. Different letters following the values indicate significant differences between treatments at *P* < 0.05. Two-way ANOVA output: ns, not significant; ^∗^*P* < 0.05, ^∗∗^*P* < 0.01. Data represent the means ± SD of three replicates.

The antioxidant enzyme activities (except MDHAR) of the mycorrhizal seedlings did not differ significantly from those of the non-mycorrhizal seedlings under well-watered control conditions (**Figures 4A–F**). Drought stress positively regulated the antioxidant enzyme activities in the watermelon seedlings. Compared with the non-mycorrhizal seedlings, the mycorrhizal plants exhibited considerably higher enzyme activities, with the exception of DHAR activity, which did not differ with AM inoculation. At the final harvest, the SOD, CAT, APX, GR, and MDHAR activities in the mycorrhizal plants were enhanced by 23.47, 24.58, 10.28, 69.49, and 25.85%, respectively, relative to the non-mycorrhizal seedlings. Interactions between watering and AM inoculation were significant for SOD (*P* < 0.05) and GR (*P* < 0.01) activities but not for the other enzymes activities. Under WW conditions, the AMF symbiosis did not result in significant differences in the non-enzymatic antioxidant contents (**Figures 4G–L**). However, the drought-induced increases in the ASA and GSH contents were more pronounced in the AM treated than in the non-AM treated plants (**Figures 4G,J**). By contrast, the induced increases in their oxidized forms (DHA and GSSG) were much lower in the former than in the latter (**Figures 4H,K**), such that the ASA/DHA and GSH/GSSG ratios were maintained at higher levels in the AM treated plants even though both plant types exhibited a decrease in these two ratios under drought stress (**Figures 4I,L**).

### Total Soluble Sugar and Proline Contents

**FIGURE 5:**
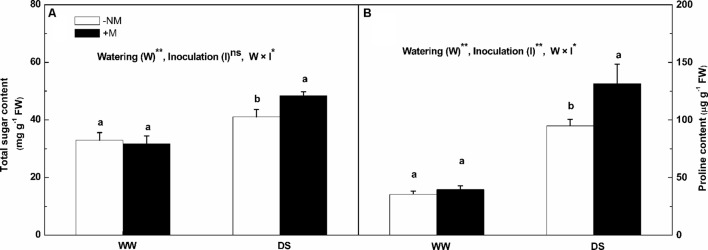
**Total soluble sugar content **(A)**, and proline content **(B)** in leaves of mycorrhizal (+M) and non-mycorrhizal (-NM) watermelon seedlings grown under WW and DS conditions**. *T*-test was performed to compare differences between inoculation treatments under the same water treatment. Different letters following the values indicate significant differences between treatments at *P* < 0.05. Two-way ANOVA output: ns, not significant; ^∗^*P* < 0.05, ^∗∗^*P* < 0.01. Data represent the means ± SD of three replicates.

The total soluble sugar and proline contents were much higher in the leaves under drought stress (**Figure 5**). The increases in the total soluble sugar and proline contents in the stressed mycorrhizal plants were more marked than in the non-mycorrhizal plants; the respective values were 52.47 and 231.26% higher than the background levels for the mycorrhizal plants compared with 24.60 and 168.48% for the non-mycorrhizal plants. Interactions between watering and AM inoculation were significant for both total soluble sugar and proline contents (*P* < 0.05).

### Relative Expression of Stress-Responsive Genes

**FIGURE 6:**
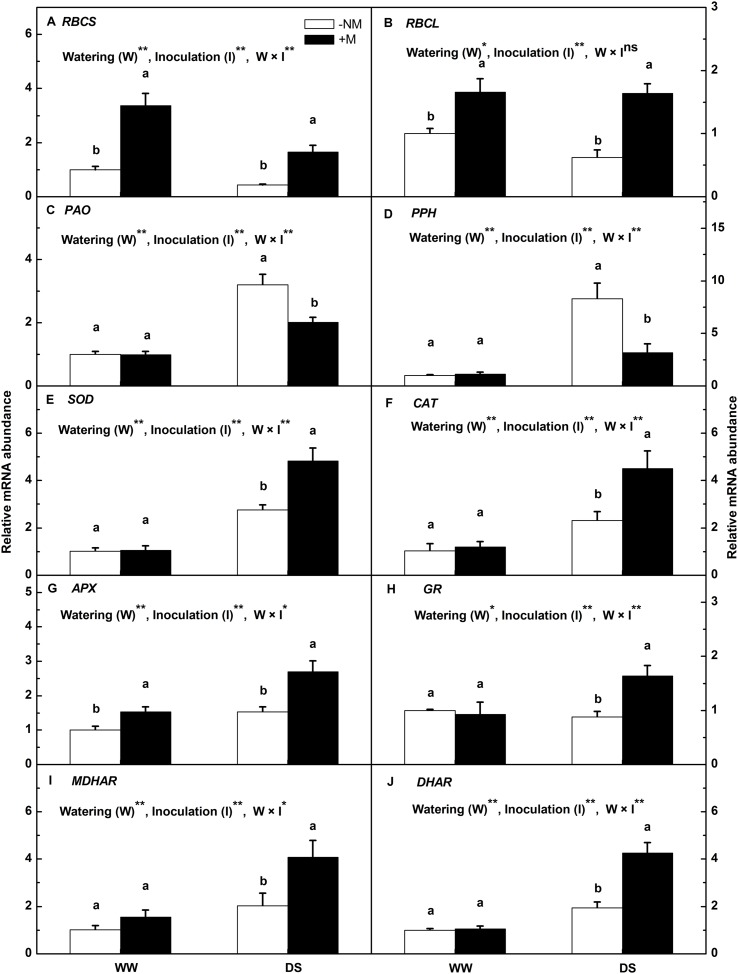
**Relative expression levels of the *RBCS***(A)**, *RBCL***(B)**, *PAO***(C)**, *PPH***(D)**, *Cu-Zn SOD***(E)**, *CAT***(F)**, *APX***(G)**, *GR***(H)**, *MDHAR***(I)**, and *DHAR***(J)** genes in mycorrhizal (+M) and non-mycorrhizal (-NM) watermelon seedlings grown under WW and DS conditions**. The expression level of the genes in the non-mycorrhizal seedlings grown under WW conditions was used as the control with a nominal value of 1. *T*-test was performed to compare differences between inoculation treatments under the same water treatment. Different letters following the values indicate significant differences between treatments at *P* < 0.05. Two-way ANOVA output: ns, not significant; ^∗^*P* < 0.05, ^∗∗^*P* < 0.01. Data represent the means ± SD of three replicates.

To gain further insights into the molecular regulation of watermelon seedlings in the presence of AM colonization, a subset of stress-responsive genes involved in photosynthesis (*RBCL* and *RBCS*), chlorophyll degradation (*PAO* and *PPH*) and the antioxidant response (*Cu-Zn SOD*, *CAT*, *APX*, *GR*, *MDHAR*, and *DHAR*) were analyzed using qRT-PCR. The results indicated that mycorrhizal inoculation significantly increased the gene expression of *RBCL* and *RBCS* regardless of treatment (**Figures 6A,B**). Compared with the well-watered control, drought stress inhibited the expression of *RBCS* and *RBCS* in watermelon plants, but with less effect on the the mycorrhizal seedlings. *PAO* and *PPH* were highly up-regulated by the drought treatment, i.e., by 3.19- and 8.28-fold in the non-mycorrhizal plants, and 2.02- and 2.81-fold, respectively, in the mycorrhizal plants (**Figures 6C,D**). Drought stress also enhanced the antioxidant gene expression; a greater enhancement was observed in the inoculated than in the non-inoculated plants (**Figures 6E–J**). The expression levels of *Cu-Zn SOD*, *CAT*, *APX*, *GR*, *MDHAR*, and *DHAR* in the DS+M plants were 1.75-, 1.93-, 1.76-, 1.85-, 2.01-, and 2.18-fold higher, respectively, than in the DS-NM plants. Interactions between watering and AM inoculation were significant for expressions of all examined genes except RBCL (*P* < 0.05).

## Discussion

In this study, we inoculated watermelon seedlings with *G. versiforme*, which is widely used to form symbiotic associations with a variety of plant species, to explore the mechanisms underlying AM-induced drought tolerance. The colonization of the watermelon roots by this AM fungus was high and not significantly affected by the restricted water supply (**Table 1**). This result differs from the findings reported by Yang Y. et al. (2014), but is in line with an observation on hybrid poplar, where drought stress did not significantly affect AM root colonization (Liu et al., 2015). We posit that this result may have been due to an insufficient time of exposure to the drought stress because the rate of colonization had already reached a certain extent before the drought treatment was imposed. Growth promotion by AM colonization has been well documented in many plant species (Huang et al., 2011; Saia et al., 2015). Here, a positive effect of AM mycorrhization on the plant morphology and growth performance of watermelon seedlings was obvious, particularly under drought stress conditions (**Figures 1E,F**; **Table 1**). Moreover, from the data presented in **Table 1**, it seems that the AM fungus played a more important role in the allocation of biomass to root growth than to shoot growth. This was further confirmed by the increased root/shoot ratio resulting from AM colonization regardless of the water regime. It has been proposed that the increased flow of photoassimilates to roots, leading to greater root system development in relation to aboveground components, not only benefits water absorption but also reduces water consumption (Toscano et al., 2014). This, coupled with the deduction that AM fungi may provide host plants additional transport channels for improving the uptake of limited water and nutrients from the soil through external hyphae (Gong et al., 2013; Yang Y. et al., 2014) might explain why under DS conditions, the mycorrhizal watermelon seedlings had a better leaf water status than the non-mycorrhizal seedlings (**Figure 2A**). Because water plays an essential role in physiological processes of plants (Gong et al., 2013), it could be deduced that the enhanced water status resulting from AM formation may assist the host plants in maintaining their normal physiological functions under drought conditions.

Photosynthesis is one of the most important physico-chemical processes of higher plants that is directly linked to plant biomass production; however, it is very sensitive to drought stress (Yang P.-M. et al., 2014). This was supported by our data, i.e., there was a considerable reduction in the *P*_n_ of watermelon seedlings under DS conditions; however, the AM symbiosis alleviated the negative effect to some extent. These findings are in good agreement with previous studies (Huang et al., 2011; Porcel et al., 2015). It has been suggested that the drought-induced suppression of photosynthesis could be generally attributed to stomatal limitation and/or non-stomatal/metabolic limitation (Zhang L. et al., 2013). Under drought stress, before any detectable changes in leaf water potential or leaf relative water content (RWC), the first response of plants is to close their stomata to minimize water loss, which is accompanied by notable decreases in stomatal conductance (*G*_s_) and *T*_r_ and consequently, stomatal limitation of photosynthesis (Flexas and Medrano, 2002; de Mezer et al., 2014). In this study, *G*_s_ (data not shown) and *T*_r_ measurements were not significantly different between the mycorrhizal and non-mycorrhizal seedlings under drought conditions (**Table 2**). Consequently, the difference in photosynthetic efficiency between these seedlings was likely caused by non-stomatal limitation rather than stomatal limitation, which is normally involved in pigment loss, deactivation of photosynthesis-related enzymes (Zhang L. et al., 2013), inhibition of the functional activity of photosystem II (PSII; Posch and Bennett, 2009), and impairment of the photosynthetic apparatus (Porcel et al., 2015). To prove this, we applied chlorophyll fluorescence techniques, which have been used extensively to accurately and harmlessly assess photosynthetic ability and energy conversion efficiency during plant responses to environmental stress (Sayed, 2003; Porcel et al., 2015). The maximum photochemical efficiency of PSII (*F*v/*F*m) reflects the potential capacity of the primary photochemistry of PSII and is usually used to reflect the degree of photoinhibition under stress conditions. Although *F*v/*F*m was inhibited by the drought treatment, this variable was significantly higher in the leaves of the DS+M watermelon plants compared with the DS-NM plants (**Table 2**). This, together with the higher ΦPSII, ETR, and qP values in the mycorrhizal plants under drought stress, implies that AM mycorrhization can sustain the efficiency of PSII photochemistry at a relatively high level. When the metabolism of a plant is disturbed by biotic or abiotic stresses, redundant energy has to be dissipated via non-photochemical processes such as heat or chlorophyll fluorescence to protect the leaf photosynthetic apparatus from photodamage (Porcel et al., 2015). The NPQ parameter is used to quantify the efficiency of heat dissipation (Sheng et al., 2008). The data collected in this study showed that the NPQ were enhanced by AM symbiosis under DS conditions (**Table 2**). The increase in NPQ suggests better avoidance of injury caused to the photosystem reaction centers and less disruption of electron transport in the photosynthetic apparatus. This was consistent with our ultramicroscopic observation (**Figure 2C**), which indicated that the chloroplast ultrastructure of the mycorrhizal watermelon leaves was better organized than that of the non-mycorrhizal leaves. Pigment content in the chloroplast is closely correlated with the photosynthetic ability of plants but easily suffers from degradation caused by drought (Porcel et al., 2015). Our data showed that the transcripts of two key genes involved in the process of chlorophyll breakdown, i.e., the *PAO* and *PPH* genes, were induced at lower levels in the AM leaves than in non-AM watermelon leaves under drought conditions (**Figures 6C,D**). Consequently, the AM watermelon seedlings experienced a lower loss of leaf chlorophyll (**Figure 2B**). Many previous studies have proved the positive effect of AM fungi on the maintenance of chlorophyll under drought stress but have not provided an actual cause. This is the first report that explains, at least partially, why AM colonization could help to inhibit chlorophyll loss of the host plants. Serving as a key enzyme in the dark reactions of the Calvin cycle, Rubisco activity and amount contribute to the extent of photosynthetic inhibition under water deficit conditions (Zhang L. et al., 2013). In the present study, drought reduced the initial Rubisco activity in the watermelon seedlings; however, the AM plants were less affected. This result is similar to the changes observed in the *RBCS* and *RBCL* gene expression (**Table 2**; **Figures 6A,B**), although the modulation of Rubisco is complicated because of post-translational modifications (Zhang L. et al., 2013). Based on the above, the less affected PSII functionality, chloroplast ultrastructural integrity, leaf chlorophyll content and initial Rubisco activity suggest that there was indeed lower metabolic limitation of photosynthesis in the AM than in the non-AM watermelon seedlings. These responses facilitated a higher photosynthetic capacity in the AM watermelon plants and improved their biomass accumulation under drought stress. The effect of AM symbiosis in alleviating the metabolic inhibition of photosynthesis has been proposed to be related to, on the one hand, the enhanced uptake and translocation of water; on the other hand, the carbon sink simulation by the presence of AM fungi for fungal carbon requirements (Porcel et al., 2015).

The enhanced production of reactive oxygen species (ROS) is a typical drought stress-derived physiological response due to the inefficient dissipation of excessive excitation energy caused by partial stomatal closure (Fan and Liu, 2011; Tian et al., 2013). A high level of ROS accumulation is deleterious to cells and results in oxidative damage (Fan and Liu, 2011). ROS scavenging is necessary and important to alleviate such oxidative stress and maintain normal plant metabolism; therefore, a highly efficient antioxidant system comprising both enzymatic and non-enzymatic antioxidants is well evolved in plant cells (Gill and Tuteja, 2010). Non-enzymatic antioxidants include low molecular metabolites, such as ASA, GSH, carotenoids (Car), and phenolic compounds (Gill and Tuteja, 2010; Boaretto et al., 2014), whereas enzymatic antioxidants include a wide range of scavenger enzymes, such as SOD, CAT, and APX, etc. (Talbi et al., 2015). Among the enzymatic antioxidants, SOD and CAT are the most common important detoxifying enzymes, which together with the cooperative enzymes (including APX, MDHAR, DHAR, and GR) in the ascorbate–glutathione (ASA-GSH) cycle, play central roles in maintaining excessive ROS under homeostatic control (Bai et al., 2010; Guo et al., 2012; Boaretto et al., 2014). In this study, all of the measured antioxidant enzyme activities were positively regulated in the watermelon seedlings to combat drought stress, and AM inoculation had a beneficial effect on this positive regulation, as manifested by the higher values of SOD, CAT, APX, GR, and MDHAR activity at both the enzymatic and transcriptional level in the stressed mycorrhizal seedlings compared with the non-mycorrhizal seedlings (**Figures 4A–F** and **6E–I**). These results confirm the findings of Ruiz-Lozano et al. (2001) and Fan and Liu (2011), who demonstrated that AM symbiosis in combination with drought stress significantly increased the enzyme activities and gene expressions involved in ROS homeostasis, providing the host plant better protection against oxidative stress, and these responses correlated well with plant tolerance to drought. It was proposed that the antioxidant protection induced by AM symbiosis is related to higher leaf Ψ as a result of AM-enhanced osmotic adjustment in AM plants (Porcel and Ruiz-Lozano, 2004), which is similar to the statement of Menconi et al. (1995), who also reported that plant water relations play an essential role in the activation and modulation of antioxidant defense systems under drought conditions. Nevertheless, the exact mechanism by which AM regulates antioxidant enzyme gene expression and activity requires more in-depth study. In the ASA-GSH cycle, in addition to the cooperative enzymes, the non-enzymatic antioxidants, i.e., ascorbate and glutathione, are also crucial in providing cellular protection by acting as substrates for the maintenance of cell redox status (Sharma and Dubey, 2005). Specifically, ASA, which functions as an electron donor, is utilized by APX to remove H_2_O_2_ (Gill and Tuteja, 2010), and GSH is responsible for regenerating ASA from its oxidized form, i.e., dehydroascorbate (Zhang W. et al., 2013), while GR, MDHAR, and DHAR help to sustain the ASA and GSH pools. Under drought stress, higher ASA and GSH contents were detected in the AM plants (**Figures 4G,J**), which coincided with greater increases in the GR and MDHAR activities (**Figures 4D–F**), implying a greater regeneration ability of non-enzymatic antioxidants to scavenge ROS in AM plants than in non-AM plants. By contrast, DHA and GSSG were less induced in the former than in the latter plants (**Figures 4H,K**); hence, higher ASA/DHA and GSH/GSSG ratios were observed in the AM-associated watermelon plants (**Figures 4I,L**), which suggests that good ascorbate and glutathione redox homeostasis was maintained in AM-pretreated plants compared with those without AM colonization. The enhanced antioxidant enzymes and the improved redox status of the cells point to more efficient antioxidant systems for ROS elimination and oxidative damage reduction activated in the AM plants in response to drought stress, as confirmed by the evidence that the MDA, H_2_O_2_, and $O_{2}^{-}$ contents increased to a lesser extent in the AM plants than in the non-AM plants when subjected to water deficit (**Figure 3**).

The drought tolerance mechanism is also related to the accumulation of osmoprotectants such as proline and soluble sugars under water shortage conditions (Mohammadkhani and Heidari, 2008). Therefore, the osmotic responses of the mycorrhizal and non-mycorrhizal plants were analyzed in this study. Under conditions of limited water supply, dramatic increases in both the total soluble sugar content and proline content were observed in the watermelon seedlings, with larger increases in the AM plants (**Figure 5**). The increased sugar content resulting from AM symbiosis may be attributed to the sink effect of the AM fungi demanding sugars from shoot tissues (Porcel and Ruiz-Lozano, 2004), while the enhanced proline accumulation via AM symbiosis is thought to be related to the up-regulation of the delta1-pyrroline-5-carboxylate synthetase gene (the rate-limiting enzyme gene in proline biosynthesis, MeP5CS; Huang et al., 2010). The result conforms to that presented by Yooyongwech et al. (2013), who reported that soluble sugar and proline, enhanced in response to AM treatment, were involved in the osmoregulatory defense response and improved the water relations in plant tissues, thereby resulting in improved growth of AM-associated macadamia plants. However, the result differs from other studies (Pinior et al., 2005; Ruiz-Sánchez et al., 2010) where the researchers proposed that the proline content might not be related to AM-induced drought tolerance. Conflicting data with respect to the effect of AM symbiosis on the osmotic adjustment of plants grown under drought conditions are likely attributable to differences in the plant and AM species used, as well as differences in the duration and severity of the drought stress.

## Conclusion

Inoculation with *G. versiforme* can enhance the drought tolerance of watermelon seedlings under limited water stress, as reflected by the improved plant growth and physiological status. The enhanced resistance of the AM-inoculated watermelon seedlings to the imposed water deficit was associated with several physiological aspects when compared with the non-AM inoculated plants, including: (1) a better developed root system for absorption of water under limited conditions; (2) improved maintenance of leaf water relations to sustain physiological metabolism; (3) a greater ability to balance energy partitioning between photochemical and non-photochemical processes, e.g., to ensure high photosynthetic capacity and to prevent the photosynthetic apparatus from being damaged; (4) a more efficient antioxidant system for ROS elimination and oxidative damage alleviation; and (5) more compatible solute accumulation to improve osmotic adjustment. All these cumulative effects of AM symbiosis ultimately increased the drought tolerance of the seedlings. Therefore, this biological strategy of establishing a symbiotic association between AM fungi and watermelon plants should be encouraged for wide application in watermelon production, especially in arid and semi-arid regions.

## Author Contributions

YM and XZ designed the study. YM, YW, RY, JZ, and CL contributed to the experiments. YM and XZ performed the data analysis. XZ, HL, JM, YZ, and CW provided guidance throughout the study. YM, YW, RY, HL, and XZ wrote and revised the manuscript. All authors approved the final manuscript.

## Conflict of Interest Statement

Theauthors declare that the research was conducted in the absence of any commercial or financial relationships that could be construed as a potential conflict of interest.

## Footnotes

1 http://www.icugi.org
