# Supplementary material for: Regulation of Plant Growth, Photosynthesis, Antioxidation and Osmosis by an Arbuscular Mycorrhizal Fungus in Watermelon Seedlings under Well-Watered and Drought Conditions
Source: Front Plant Sci. 2016 May 11;7:644. doi: 10.3389/fpls.2016.00644 (PMC4862978; doi:10.3389/fpls.2016.00644)
Supplement: Supplementary file 1 [file Table_1.DOC]

**SUPPLEMENTARY TABLE**

**TABLE S1 | The primers used for real-time RT-PCR.**

| **Gene** | **Gene ID** | **Primer sequence (5’-3’)** | **Gene description** |
| --- | --- | --- | --- |
| RBCS | Cla020400 | F: GTGATTCAGGAGATTGAGGAG | Rubisco small subunit |
|  |  | R: GTAGAACTTAGGGGGCTTG |  |
| RBCL | Cla003384 | F: CACCACAAACAGAGACTAAAGCA | Rubisco large subunit |
|  |  | R: TCGGAATGCTGCCAAGATAT |  |
| PAO | Cla001857 | F: CGAGCCAATGCCACTACAC | Pheide a oxygenase |
|  |  | R: TATCCCGCCTCCCTGTAAC |  |
| PPH | Cla015204 | F: GATATGGGACGTGATTACAGAG | Pheophytin pheophorbide hydrolase |
|  |  | R: GATGCTGCCATAAGTCAATAGAG |  |
| Cu-Zn SOD | Cla008698 | F: AGCCATTGTAGATACCCAGATTC | Cu-Zn subunit-superoxide dismutase |
|  |  | R: CTGAGTTCGTGACCTCCTTT |  |
| CAT | Cla023447 | F: ACTTGTGCCGATTTCCTTCG | Catalase |
|  |  | R: ATTGCCCTCCCTGGTGTAA |  |
| cAPX | Cla022327 | F: GGGAAGTTGAACGGATTAGAG | Cytoplasmic ascorbate peroxidase |
|  |  | R: CAGCATCGTGAAATACCAGAC |  |
| cGR | Cla021430 | F: GGAGTCGCTGTCGCTCTTAA | Cytoplasmic glutathione reductase |
|  |  | R: CTACCTGCTTCAATTCGCCT |  |
| MDHAR | Cla018379 | F: CTGGATATGCGGCTAGGGA | Monodehydroascorbate reductase |
|  |  | R: GTGACTCGGGAAAGAGATAG |  |
| DHAR | Cla020113 | F: AAGCAAGGACCCAAATGATG | Dehydroascorbate reductase |
|  |  | R: TGGTGCCAGGCTCAAATCA |  |
